# Dissociable effects of music and white noise on conflict-induced behavioral adjustments

**DOI:** 10.3389/fnins.2022.858576

**Published:** 2022-08-17

**Authors:** Alexander J. Pascoe, Zakia Z. Haque, Ranshikha Samandra, Daniel J. Fehring, Farshad A. Mansouri

**Affiliations:** ^1^Cognitive Neuroscience Laboratory, Department of Physiology, Monash Biomedicine Discovery Institute, Monash University, Clayton, VIC, Australia; ^2^ARC Centre of Excellence for Integrative Brain Function, Monash University, Clayton, VIC, Australia

**Keywords:** executive control, conflict processing, music, white noise, Stroop test, online testing

## Abstract

Auditory stimuli, encompassing a continually expanding collection of musical genres and sonic hues, present a safe and easily administrable therapeutic option for alleviating cognitive deficits associated with neuropsychological disorders, but their effects on executive control are yet to be completely understood. To better understand how the processing of certain acoustic properties can influence conflict processing, we had a large of cohort of undergraduate students complete the Stroop colour and word test in three different background conditions: classical music, white noise, and silence. Because of pandemic guidelines and the necessity to run the experiment remotely, participants also completed the Wisconsin card sorting test (WCST), so that the reliability and consistency of acquired data could be assessed. We found that white noise, but not classical music increased the response time difference between congruent (low conflict) and incongruent (high conflict) trials (conflict cost), hence impairing performance. Results from the WCST indicated that home-based data collection was reliable, replicating a performance bias reported in our previous laboratory-based experiments. Both the auditory stimuli were played at a similar intensity, thus their dissociable effects may have resulted from differing emotional responses within participants, where white noise, but not music elicited a negative response. Integrated with previous literature, our findings indicate that outside of changes in tempo and valence, classical music does not affect cognitive functions associated with conflict processing, whilst white noise impairs these functions in a manner similar to other stressors, and hence requires further research before its implementation into neuropsychiatric care.

## Introduction

The ability to efficiently process conflicting information is a core feature of executive control (Miller and Cohen, 2001; Mansouri et al., 2020b), and is believed to recruit a wide network of prefrontal regions (Botvinick et al., 2001; Kerns et al., 2004; Mansouri et al., 2017b,^2022^; Mansouri and Buckley, 2018). The colour-word matching variant of the Stroop test offers an easily reproducible method of assessing an individual’s ability to separate information pertaining to colour and word meaning (Stroop, 1935; MacLeod and MacDonald, 2000; Zysset et al., 2001). Deficits in Stroop performance have been observed in several psychiatric disorders (Bannon et al., 2002; Lansbergen et al., 2007; Kravariti et al., 2009; Joyal et al., 2019), which suggests impaired conflict processing may underlie some of their symptoms. Current treatment options for these conditions are limited, consisting primarily of psychotherapy or pharmacological interventions, which generally have poor efficacy rates (Thase, 2007; Kirsch et al., 2008; Fournier et al., 2010; Stafford et al., 2015; Cuijpers et al., 2018), waning compliance (Velligan et al., 2009; Semahegn et al., 2020), and/or a myriad of potential debilitating side effects (Henderson, 2008; Serretti et al., 2013; Hilt et al., 2014; Stafford et al., 2015). Therefore, it is evident there is a need for additional treatment options that are both safe, and easily administrable to alleviate the cognitive deficits associated with these conditions. Changing the context in which information processing takes place provides a relatively simple avenue for potentially modulating performance in various cognitive domains. Background auditory stimuli, ranging from various genres of music to differing power spectrums of noise signals (sonic hues), are some of the most commonly explored contextual factors, yet their effects on cognitive processes remain inconclusive, with all of positive (Miller and Schyb, 1989; Day et al., 2009; Manan et al., 2012; Masataka and Perlovsky, 2013; Rausch et al., 2014; Proverbio et al., 2015; Angwin et al., 2017; Feizpour et al., 2018; Othman et al., 2019), null (Kämpfe et al., 2010; Wais and Gazzaley, 2011; Jing et al., 2012; Bottiroli et al., 2014; Jäncke et al., 2014; Herweg and Bunzeck, 2015; Kou et al., 2017; Lehmann and Seufert, 2017; Burkhard et al., 2018; Fehring et al., 2019b), and negative (Brodsky, 2001; Furnham and Strbac, 2002; Cassidy and MacDonald, 2007; Dobbs et al., 2011; Jing et al., 2012; Brodsky and Slor, 2013; Masataka and Perlovsky, 2013; Proverbio et al., 2015; Musliu et al., 2017; Feizpour et al., 2018; Cloutier et al., 2020) outcomes having been reported in cognitive task performance for healthy populations. Underlying these differences may be significant variation in study designs (between-subject or within-subject), presented stimuli, and cognitive tasks. Significant research is necessary to understand these distinctions, and thus determine their suitability for use within neuropsychiatric care.

One of the foremost difficulties in studying music is its conceptual broadness, which has particularly expanded in the past century with the rapid development of new musical styles and genres. From a quantifiable perspective, music can be differentiated from other sounds by the pitch, loudness, and timbre of its constituent tones, and the ordered timing (rhythms) in which these tones are arranged (Roederer, 2008). In a more psychological context, music has been considered to relate to appetitive urges, consummatory expression, drive, and satisfaction; allowing the communication of these bodily states and information among members of the same species through the modulation of each other’s emotional states (Dewey, 1958). Research into music’s effects on physiology has since identified that the valence (e.g., joyous/smooth, sad/harsh) and tempo (slow, fast) of songs can induce dissociable effects in measures of emotional state, and related cortical activation (Blood et al., 1999; Schmidt and Trainor, 2001; Carpentier and Potter, 2007; Arjmand et al., 2017; Mansouri et al., 2017a). Accordingly, behavioural studies have centred on describing how these emotional alterations can affect cognitive task performance, generally making comparisons between different music conditions (e.g., positive vs. negative valence, slow vs. fast tempo), and to silence. Although such research has proven insightful, fewer behavioural studies have included other, task-irrelevant sounds in their designs, where those having done so have often reported contrasting outcomes depending on the relevant task (Furnham and Strbac, 2002; Bottiroli et al., 2014), or musical genre/type (Cassidy and MacDonald, 2007; Bottiroli et al., 2014; Proverbio et al., 2015). Thus, whilst it is well established that music can alter an individual’s emotional state (and subsequently their higher cognitive function, albeit with various uncertainties/task-dependencies) through variation in valence and tempo, it remains unclear if there exists dissociable effects on executive control, when compared to silence, between music and other non-musical sounds. Considering results from previous studies have differed between genres, classical music could be considered suitable for continuing such investigations, as tempo and valence can vary considerably between songs, it is often non-lyrical, and contains a wide array of rhythmic patterns and melodies. Whilst classical genres have been extensively used in behavioural research (particularly in investigating the “Mozart effect”), most studies use only select songs with an identifiable valence or tempo, rather than a varied/expansive playlist. Therefore, we postulated that by playing a wide and varied collection of classical songs, any emotional alterations produced from the valence and/or tempo of songs would fluctuate, and hence not significantly affect overall performance. Inclusion of another task-irrelevant, non-musical sound in our design would then allow further understanding of the interaction between executive control and the perception of music.

Outside of music, one of the most commonly used auditory stimuli in neuroscience research is white noise, which consists of sound at every frequency of the human hearing rage (20 Hz–20 kHz) played at equal intensities. Its use within the field primarily stems from the phenomenon of stochastic resonance, where moderate levels of random noise can improve information transfer and processing in man-made and naturally occurring non-linear systems (Moss et al., 2004; McDonnell and Abbott, 2009). In the context of neural processing, it was originally found the addition of white noise at moderate levels could improve the detection of proprioceptive, tactile, and visual stimuli (Manjarrez et al., 2007; Lugo et al., 2008). Electroencephalography (EEG) examination subsequently revealed that white noise can improve neural synchronisation within and between brain regions, expanding to regions typically distinguished from sensory processing such as the superior frontal gyrus and posterior cingulate cortex (Ward et al., 2010). Behavioural research has since shown that low (50–60 dB) and moderate (70–80 dB) levels of white noise can improve performance in auditory working memory and semantic memory tasks (Manan et al., 2012; Rausch et al., 2014; Herweg and Bunzeck, 2015; Angwin et al., 2017; Othman et al., 2019), with functional magnetic resonance imaging (fMRI) taken during performance of these tasks finding increased blood oxygen level dependent (BOLD) signal in various cortical, midbrain, and brainstem regions (Manan et al., 2012; Rausch et al., 2014; Othman et al., 2019). However, performance in other cognitive domains has failed to replicate these benefits, with outcomes in visual working memory, set-shifting, and phonemic fluency tasks unchanged (Bottiroli et al., 2014; Herweg and Bunzeck, 2015), or even impaired (Herweg and Bunzeck, 2015). Moreover, investigations in school children have found improvements only in those diagnosed with attention-deficit/hyperactivity disorder (ADHD) and not healthy comparisons (Söderlund et al., 2007, 2010, 2016). These contrasting findings suggest any cognitive facilitation provided by white noise may be sensitive to both differences between tasks, and study cohorts.

Considering white noise is beginning to be implemented into healthcare and research environments for a variety of purposes (Patterson-Kane and Farnworth, 2006; Farokhnezhad Afshar et al., 2016), including in patients suffering from neuropsychological disorders (Kaneko et al., 2013; Lin et al., 2018), the factors underlying these distinctions must be better understood. One outstanding question is whether processes underlying executive control can benefit from white noise, where there exist contrasting findings between working memory tasks (Manan et al., 2012; Bottiroli et al., 2014; Herweg and Bunzeck, 2015; Othman et al., 2019), hence highlighting a need for a greater variety of executive functions to be tested in its presence. Because improvements from white noise have indicated relatively small effect sizes (Manan et al., 2012; Rausch et al., 2014; Herweg and Bunzeck, 2015; Angwin et al., 2017; Othman et al., 2019), it is also necessary to consider the sensitivity of related outcome measures in conducting such testing. The Stroop test, which under the umbrella of conflict processing assesses cognitive flexibility, response inhibition, and selective attention (Grandjean et al., 2012; Scarpina and Tagini, 2017), as well as containing sensitive outcome measures such as conflict cost and adaptation (Gratton et al., 1992; Botvinick et al., 1999, 2004; Mayr et al., 2003; Carter and van Veen, 2007; Mansouri et al., 2017b,^2022^), satisfies these criteria, and as such may be appropriate for better understanding the parameters of white noise’s influence on executive control.

Due to pandemic-related social distancing orders, we had to reformat our planned experiment to a home-based study, which has remained a relatively unexplored area in psychophysics research (Semmelmann and Weigelt, 2017). For accessibility to participants, we decided to use a well-established and reliable platform for online behavioural and cognitive testing paradigms, PsyToolkit, which has been developed by a researcher within the field (Stoet, 2010, 2016). Whilst our change to the platform was forced due to the COVID pandemic, the use and advancement of remote testing within psychophysics may prove beneficial for mobility-limited or geographically isolated individuals, such as the elderly or those living outside major metropolitan areas. The primary concern surrounding these external platforms is the reliability of sensitive measures such as response time, and adherence to experimental protocols and procedures. Accounting for these factors, we included an analogue of the Wisconsin card sorting test (WCST) into our design, where a response time bias to colour-matching over shape-matching has been consistently reported in previous studies (Mansouri et al., 2020a,b). Because both version of the WCST (laboratory-based and home-based) included both colour and shape matching, and the two cohorts were similar in background (age, education), we could compare these biases between studies to assess the sensitivity and reliability of remote data acquisition.

**FIGURE 1:**
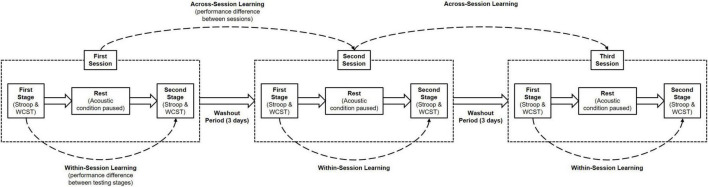
Experimental protocol for assessing the effects of background acoustic conditions on cognitive functions. Participants performed both the Stroop test and Wisconsin card sorting test in three separate daily sessions, each separated by a 3-day washout period. Within a daily session there were two testing stages, where each test was completed once over, separated by a ten-minute rest period. A different background acoustic condition was played in each daily session. This protocol allows for the assessment of within-session learning (the behavioural changes occurring between the first and second testing stages in the same daily session). The order of both background acoustic conditions and task performance was counterbalanced across the three daily sessions, accounting for the influence of across-session learning.

Therefore, to further explore the effects exerted by background acoustic stimuli within the context of conflict processing, we designed a three-session, two-staged, repeated-measures experiment using both the Stroop test and WCST (Figure 1). In each session, participants were to perform the tests in the presence of a varied and expansive classical playlist, white noise, or silence. Both tests were readily available for use and modification through the PsyToolkit experiment library. The inclusion of a pre-post design allowed for the consideration of within-session learning effects, which have been reported in previous psychophysical studies (Fehring et al., 2019a,^2022^), and may even be modulated by contextual factors during conflict processing (Fehring et al., 2019b). The order in which background acoustic stimuli were presented was counterbalanced to offset any influence of across-session learning, and the order of task performance was also counterbalanced to negate any potential advantage or disadvantage of performing one test before the other.

In using classical music and white noise, our study design encompassed a great breadth of acoustic properties; from complex rhythmic combinations and intricate melodies, to a random distribution of sound within each frequency of the Human hearing range (20Hz-20kHz), and hence their investigation may serve as a foundation for enquiry into the higher-order cognitive effects of more nuanced acoustic stimuli. This design compliments current literature regarding background auditory stimuli and the Stroop test, which have centred around certain musical types (high/low tempo, positive/negative valence) and more naturalistic noises (a range of traffic, office, and voice-related sounds) (Cassidy and MacDonald, 2007; Masataka and Perlovsky, 2013). Due to such significant differences between the stimuli, we hypothesised that they may differentially affect participant’s conflict processing capabilities. In particular, we expected white noise may impair Stroop performance, given the sensitivity of the tests outcome measures, combined with the neutral (Bottiroli et al., 2014; Herweg and Bunzeck, 2015) and negative (Herweg and Bunzeck, 2015) results reported in other visual-based tasks assessing aspects of executive control. Overall, we aimed to further elucidate the interaction between auditory processing and executive control processes, alongside helping to explore new effective, safe, and well-tolerated treatments for cognitive deficits associated with neuropsychological disorders.

## Materials and methods

### Participants

Sixty-seven Monash University undergraduate students (45 females, 22 males) chose to participate in the project as part of their coursework. *A priori* power analysis was performed based on the effect size observed in our previous studies in which we examined the effects of background acoustic conditions (music) in the context of cognitive tasks (Feizpour et al., 2018). With the significance level of 0.05 and the power at 0.80, the estimated sample size for this study was 61 participants (using G*Power 3.1; Faul et al., 2007). However, 67 participants allowed for full counterbalancing and maintenance of an adequate sample in the circumstance some participants could not complete their data collection. Participants were instructed to complete the Stroop test and WCST at home (during the COVID-related lockdown period in 2020) in three different background acoustic conditions, each separated by at least three days. The order of acoustic conditions and cognitive tests (Stroop/WCST) was counterbalanced. We also aimed to counterbalance the number of males and females in each condition; however, due to the uneven sex ratio we could not achieve a perfect equilibrium. All participants were between the ages of 18 and 29 (21.06 ± 1.82; mean ± standard error), with similar educational level (third-year University science students), and had no history of any neurological disorders, nor any medical conditions that may interfere with performing the tests or listening to the acoustic stimuli (checked by a screening questionnaire). Approval was obtained from the Monash University Human Research Ethics Committee. Informed consent was obtained from all participants.

### Home-based experiments

To facilitate remote testing, we used PsyToolkit, a free web-based service that can be edited to run various cognitive tests and accessed from participants’ personal computers (Stoet, 2016). The programs within PsyToolkit use an efficient Linux-based scripting language which allows for millisecond timing precision, as is required when recording response times in cognitive tests (Stoet, 2010). This online platform for behavioural testing has been developed and validated by neuroscientists (Stoet, 2010, 2016).

Participants were provided with clear instructions (explanatory written documents for setting the hardware, software, and two online tutorial sessions) which detailed how they should prepare for and conduct the two tasks. This included keeping time of day (which was the quietest possible time), distance from their monitor, and mouse position constant, the intensity level they should use among acoustic conditions, turning off mobile phones, closing other programs, and alerting any household members they are not to be disturbed during the session. After completing each task, participants were instructed to copy the resulting data output into a provided spreadsheet, and at the end of their three testing sessions, they sent this spreadsheet *via* email to investigators. A detailed explanation of how the experiment and related classes were run can be found in Jaberzadeh and Mansouri (2021).

### Procedure

Each of the three daily sessions required participants to complete both tests twice, with a 10-min break between their repetitions (Figure 1). Participants were told they could pause the current acoustic condition and have a drink of water or juice (no caffeinated or alcoholic beverages) during this break period. As a whole, sessions should have lasted no longer than one hour, with 70 Stroop trials and 60 WCST trials required for completion each time. Information regarding the PsyToolkit WCST can be found in the Supplementary material (Appendix 1) and Supplementary Figure 1.

#### PsyToolkit Stroop test

**FIGURE 2:**
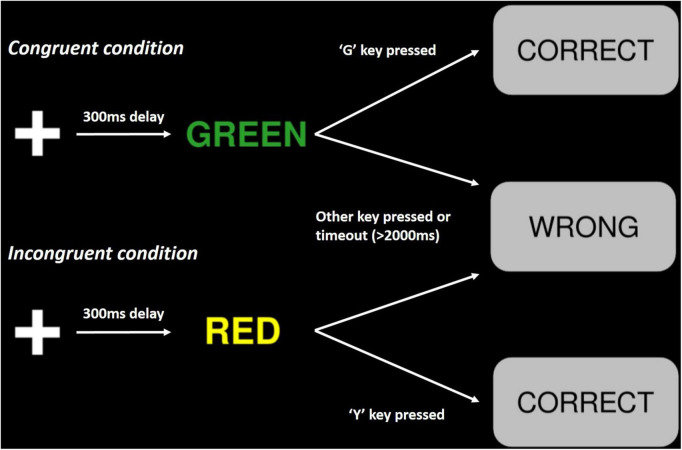
Schematic representation of the experimental paradigm in the PsyToolkit Stroop test. Trials begin with on the onset of a white cross, followed by the presentation of a colour name printed in coloured ink. A grey text box displaying either “CORRECT” or “WRONG” indicates a correct or incorrect response, respectively (Stoet, 2010, 2016).

The PsyToolkit variant of the Stroop test (Figure 2) is mostly analogous to the standard colour-word matching Stroop test. Trials begin with the presentation of a white cross against a black background, which after 300 ms is replaced by the name of a colour printed in coloured ink. Participants are instructed to respond to the print colour, which can be the same (congruent) or different (incongruent) to the colour name, by pressing the corresponding key on their keyboard. These keys are “R” for red, “Y” for yellow, “G” for green, and “B” for blue. Feedback is delivered to participants by the appearance of a grey box, with text consisting of “CORRECT” or “WRONG.” This textbox lasts for 500 ms, before the commencement of the following trial. If participants fail to respond within 2,000 ms following word presentation, their response is recorded as a “timeout.” Participants performed 70 trials each testing stage; 15–30% of these trials were congruent (low congruent), and 70–85% were incongruent (high conflict).

### Acoustic conditions

Participants were provided with links to download the required acoustic conditions and therefore all participants listened to the same set of songs and white noise. They were instructed to play these songs or files by their preferred medium, whether earphones, headphones, or speakers, at a moderate intensity where they could hear the stimuli clearly, but not have it bother them; similar to how they would normally listen to music. It was also emphasised that this intensity level should remain constant between the two stimuli (music and white noise), and that their attention remain solely on the cognitive task. There was no particular sound level set across participants, but instead participants were instructed (both in the preceding classes and written instruction) to listen to both acoustic stimuli at a level similar to that they would listen to music; a moderate intensity where they can hear the stimuli clearly without being annoyed by loud sound. It is important to consider some individuals may be accustomed to listening to music and other auditory stimuli at higher intensities than others, and therefore it may be beneficial to have participants choose their own definition of moderate. In our previous laboratory-based experiments, we have also allowed participants to adjust volume levels to their preference (Mansouri et al., 2017a; Feizpour et al., 2018; Fehring et al., 2019) because some participants found pre-set intensities to be too loud or inaudible. Setting a particular sound intensity for all participants might lead to non-specific effects, such as being annoyed by loud sound, and become a confounding factor.

For the music condition, a free online playlist, *via* archive.org (a non-profit digital library) was provided; “100 Classical Music Masterpieces.” This playlist was chosen as it includes predominantly instrumental pieces dating from 1685 to 1928, with no distinct preference to a given valence or tempo between songs. A full list of the included songs can be found in the Supplementary material (Appendix 3). For the white noise condition, a MP3 audio file, which encoded 2 h of continuous white noise (20–20 kHz, same intensity throughout), was provided to participants. This file was generated using Audacity, a free open-source audio editor and recorder.

### Data analyses

Repeated-measures ANOVAs were used to assess the effects of practice, trial type (conflict level or rule), background acoustic condition, and sex on various behavioural measures. Within-session practice-related learning could be assessed due to the two-stage design, where each test was completed twice per session. Including Practice and Sex factors into the repeated-measures ANOVAs was necessary considering they have both been found to influence performance in several cognitive tasks (Mansouri et al., 2016b; Feizpour et al., 2018; Fehring et al., 2019a,^2021^, ^2022^), and may also interact differentially with background acoustic conditions (Feizpour et al., 2018). For the Stroop test, response time was measured as the time from presentation of the colour name to the registration of keyboard input. For the WCST, response time was measured as the time from trial onset to registration of a mouse click on one of four target items. The timeout thresholds for Stroop and WCST trials were 2,000 and 10,000 ms, respectively, therefore all response time data were limited to these ranges. Implementing arbitrary procedures for removing outlying data points may bias results and outcome of statistical analyses, thus we included all data points in the statistical analyses. Considering the variance in mean response time between participants, and in order to ease comparison of testing stages and acoustic conditions, response time data was normalised by dividing each value by the grand average for all conditions in each individual. This normalisation procedure has been implemented in previous behavioural studies (Mansouri et al., 2016a,2017a,2020a; Fehring et al., 2019b,a, 2022). Response accuracy for both tests was calculated as the percentage of correct trials (correct responses/total responses), and was analysed without any normalisation. Normalisation was not required as there was a high level of accuracy (majority > 80%) and consistency across all stages and conditions in both tests.

Three participants, however, were excluded from analyses. One was taking prescribed psychoactive medication and reported feeling ill during two sessions, whilst for the other two the results were incomplete. Therefore, in total results from 64 participants, 43 females and 21 males, were included in the analyses. Using repeated-measures ANOVAs, including predominantly within-subject factors, reduces the effects of non-specific factors such as sleep level, food-drink, and emotional or motivational state. The order of cognitive tasks and acoustic conditions were counterbalanced to control for any confounding effect of across-session learning or its interaction with other factors.

Sphericity was examined (Mauchly’s test) for all ANOVA measures and if violated, Greenhouse-Geisser correction was implemented. Significance level was set at 0.05 for all statistical tests. For significant effects, partial eta squared (η_p_^2^) is reported, which indicates the proportion of variance explained by the effect in the ANOVA analysis. Where significant interactions were detected, pairwise comparisons were conducted. Pairwise comparisons consisted of a two-tailed *t*-test with Bonferroni adjustment for multiple comparisons.

## Results

### Stroop test

Within-session practice-related learning, differing levels of conflict, and acoustic environment may interactively affect cognitive control, and consequently performance in cognitive tasks. To examine the interaction between these factors and conflict processing, a multifactorial repeated-measures ANOVA was applied to the mean normalised response time and mean percentage of correct trials. The ANOVA included Practice (first/second stage), Conflict (congruent/incongruent), and Sound (music/noise/silence) as within-subject factors, and Sex (female/male) as a between-subject factor.

#### Acoustic environment and conflict level interactively modulated performance

**FIGURE 3:**
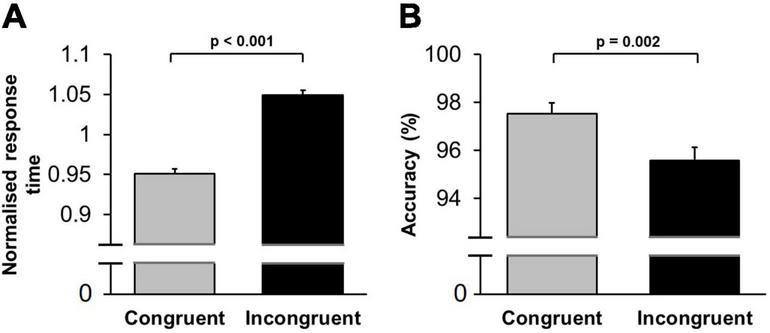
Conflict-induced behavioural adjustment in the Stroop test. **(A)** Mean normalised response time is shown for congruent and incongruent trials. Response time was significantly shorter in low conflict (congruent trials), compared to high conflict (incongruent trials). **(B)** The mean percentage of correct responses (accuracy) is shown for congruent and incongruent trials. Accuracy was significantly higher in congruent trials compared to incongruent trials. Error bars represent standard error of the mean (SEM).

**FIGURE 4:**
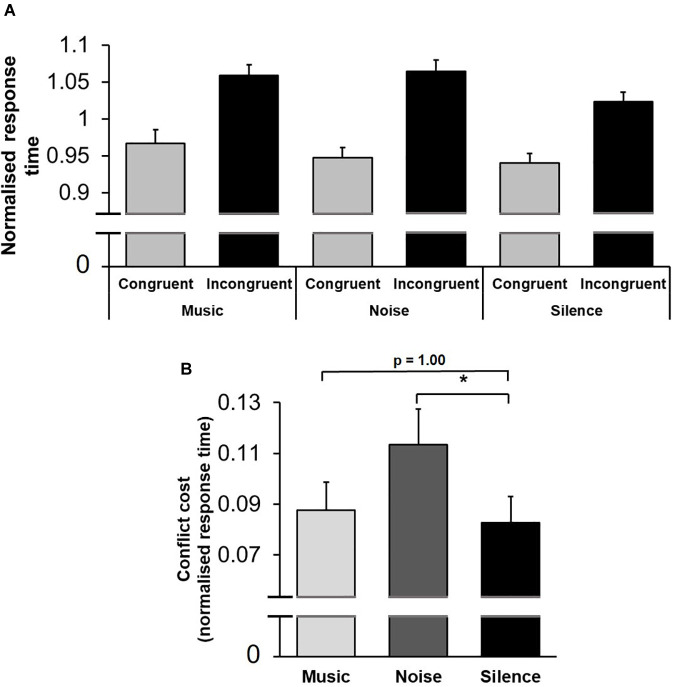
Conflict cost was modulated by the background acoustic condition. **(A)** Mean normalised response time is shown for congruent and incongruent trials in each background acoustic condition. The acoustic environment modulated the conflict-induced alterations in response time. **(B)** The difference in mean normalised response time between congruent and incongruent trials (conflict cost) is shown for each background acoustic condition. Compared to silence, white noise increased the conflict cost. *represents *p* < 0.05. Error bars represent standard error of the mean (SEM).

A multifactorial repeated-measures ANOVA [Practice (first/second stage of testing, within-subject factor) × Conflict (congruent/incongruent trials, within-subject factor) × Sound (music/noise/silence, within-subject factor) × Sex (female/male, between-subject factor)] applied to mean normalised response time revealed a significant main effect of Practice [*F*(1,64) = 78.86; *p* < 0.001, η_p_^2^ = 0.56]: response time decreased from the first to second stage of testing within the same session (within-session learning). The main effect of Conflict was highly significant [*F*(1,64) = 74.10; *p* < 0.001, η_p_^2^ = 0.54]: response time was longer in incongruent trials (Figure 3A). Importantly, the main effect of Sound was not significant [*F*(2,128) = 1.10; *p* = 0.34]: background acoustic condition did not influence overall response time. There was, however, a significant interaction between Conflict and Sound factors, [*F*(2,128) = 3.23; *p* = 0.043, η_p_^2^ = 0.050], indicating that the background acoustic condition differentially affected response time depending on the level of conflict encountered (Figure 4A). We also performed the same multi-factorial repeated-measures ANOVA on the raw (non-normalised) response time values. Similar to the results from the normalised data, there were significant main effects for Practice [*F*(1,64) = 61.89; *p* < 0.001, η_p_^2^ = 0.50] and Conflict [*F*(1,64) = 57.81; *p* < 0.001, η_p_^2^ = 0.48], and a significant interaction between Conflict and Sound factors [*F*(2,128) = 3.15; *p* = 0.046, η_p_^2^ = 0.048], whilst the main effect of Sound was not significant [*F*(2,128) = 1.01; *p* = 0.37].

Conflict cost refers to the difference in performance between congruent and incongruent trials, reflecting the higher processing demands for resolving conflict between competing options (Botvinick et al., 2004; Carter and van Veen, 2007; Mansouri et al., 2017b). To further investigate the interaction of Conflict and Sound factors, conflict cost was calculated by subtracting mean response time in congruent trials from those in incongruent trials for each sound condition. In a planned comparison, the conflict cost for music and noise conditions were contrasted with that of silence in separate pairwise comparisons (paired two-tailed *t*-test with Bonferroni adjustment for multiple comparisons). The conflict cost was not significantly different between the music and silence conditions [t(63) = 0.38; *p* = 1.00]; however, conflict cost was significantly different between noise and silence conditions (t(63) = 2.52; *p* = 0.029]. This indicates that the conflict cost in the presence of white noise was increased (Figure 4B). The same paired two-tailed *t*-tests performed using the conflict cost calculated from non-normalised response time values returned similar results, where the difference between music and silence was not significant [t(63) = 0.0052; *p* = 1.00], whilst the difference between noise and silence was significant [t(63) = 2.67; *p* = 0.019].

The same multifactorial repeated-measures ANOVA [Practice (first/second stage of testing, within-subject factor) × Conflict (congruent/incongruent trials, within-subject factor) × Sound (music/noise/silence, within-subject factor) × Sex (female/male, between-subject factor)] applied to mean percentage of correct trials showed an insignificant main effect of Practice [*F*(1,64) = 0.41, *p* = 0.52, η_p_^2^ = 0.007]: accuracy did not change between the first and second testing stages of each session. Meanwhile, the main effect of Conflict was significant [*F*(1,64) = 11.00, *p* = 0.002, η_p_^2^ = 0.15]: accuracy was lower in incongruent trials (Figure 3B). The main effect of Sound was not significant [*F*(2,128) = 0.54; *p* = 0.58]: background acoustic condition did not change accuracy. The interaction between Sound and Conflict factors was also not significant [*F*(2,128) = 13.68; *p* = 0.56], indicating that accuracy in both conflict conditions was equally unaffected by the background acoustic condition.

#### Acoustic environment did not influence conflict adaptation

The effects of conflict on performance are not limited to the current trial in which the conflict is experienced, but can also be observed in the subsequent trial, where a behavioural improvement can occur if the subject is presented with a high level of conflict again. This conflict-induced behavioural change is known as conflict adaptation (Gratton et al., 1992; Botvinick et al., 1999; Mayr et al., 2003; Mansouri and Buckley, 2018; Mansouri et al., 2022). In the context of the Stroop test, it can be examined through contrasting incongruent trials that were immediately preceded by another incongruent trial (ii sequence) to those preceded by a congruent trial (ci sequence). Therefore, to detect if conflict adaptation was evident in this study, and if acoustic environment modulated its effect, we applied a repeated-measures ANOVA to mean normalised response time in the second trial of each sequence (ci/ii). The multifactorial repeated-measures ANOVA [Practice (first/second stage of testing, within-subject factor) × Trial Sequence (ci/ii, within-subject factor) × Sound (music/noise/silence, within-subject factor) × Sex (female/male, between-subject factor)] applied to mean normalised response time in the second trial of each sequence revealed a significant main effect of Trial Sequence [*F*(1,64) = 6.33; *p* = 0.014, η_p_^2^ = 0.093]: response time was shorter in ii sequences. Notably, the interaction between Sound and Trial Sequence factors was not significant [*F*(2,128) = 0.76; *p* = 0.47, η_p_^2^ = 0.012], indicating that acoustic environment did not influence conflict adaptation. Performing an identical multifactorial repeated-measure ANOVA using non-normalised response time values returned alike results, where the main effect of Trial Sequence was significant [*F*(1,64) = 6.017, *p* = 0.017, η_p_^2^ = 0.088], and the interaction between Sound and Trial factors was insignificant [*F*(2,128) = 0.71; *p* = 0.49, η_p_^2^ = 0.011].

### Wisconsin card sorting test

In this study, participants performed both Stroop test and the WCST. Inclusion of the WCST allowed for the assessment of reliability and sensitivity of the measurements in remotely collected data by comparison of results with those obtained in our laboratory-based studies (Mansouri et al., 2020a). One of the consistent findings in our laboratory-based testing with a computerised WCST is that young adults show a significant behavioural bias to colour matching over shape matching, which appears as a shorter response time and higher accuracy in the colour-matching blocks (Mansouri et al., 2020a,b). Therefore, to examine whether this bias could also be detected in home-based studies, we applied a multifactorial repeated-measures ANOVA to mean normalised response time in correct trials and mean percentage of correct trials. The ANOVA included Practice (first/second stage), Rule (colour/shape/number), and Sound (music/noise/silence) as within-subject factors, and Sex (female/male) as a between-subject factor. We found that the previously reported dimensional bias in laboratory settings was replicated in this cohort tested using an online platform (Supplementary Figures 2A,B). For further information about these results, please see the Supplementary material (Appendix 1).

## Discussion

The aim of this study was to investigate how potential cognitive effects produced by background music and white noise may influence conflict processing. Our findings indicate that in the context of the Stroop test, white noise, but not classical music significantly affected the conflict cost (the difference in response time between congruent and incongruent trials), whereby participant’s ability to resolve conflict was impaired (Figure 4B). These results provide important reference for previous behavioural studies assessing both background music and noise (Furnham and Strbac, 2002; Cassidy and MacDonald, 2007; Bottiroli et al., 2014; Proverbio et al., 2015), where even when a large, varied playlist of complex music is employed, thus reducing the influence of emotionally salient properties within songs such as valence and tempo, and noise is made to be stochastic (white noise), there still exists a dissociable influence between the stimuli on a core component of executive control (conflict processing) when compared to silence. Remarkably, such findings are also homologous with a previous study in a non-human primate species (macaque monkeys) where similar, dissociable task-dependent effects between classical music and white noise were reported (Zarei et al., 2019; Supplementary material Appendix 2). Additionally, because of the necessity to run the experiment remotely, we assessed the reliability of remote data collection by comparing rule-based performance differences in the WCST between the present (home-based), and one of our previous laboratory-based studies. In the home-based study, we found a significant bias toward the colour dimension over shape, which replicates consistent observations made in our laboratory-based experiments with a comparable version of the WCST (Supplementary Figures 2A,B). Below, we discuss the implications of these findings, and how they can help to clarify outstanding questions in the current literature.

### Music had no effect on conflict processing

By using a varied playlist of classical music and white noise, we sought to investigate how music and white noise may differentially affect executive control when compared to silence. In the Stroop test, background music did not significantly alter participant’s response time or accuracy, nor did it modulate the conflict cost or conflict adaptation effects. This absence of effect contrasts white noise, which increased the conflict cost, indicating that the processing of each stimuli lead to dissociable effects that became apparent when participants had to resolve conflict (incongruent trials). Considering individuals have been shown to consistently rank various noises lower in scales of emotional preference compared to musical excerpts (Gomez and Danuser, 2004), it is possible that a negative emotional state was induced by the white noise, but not the classical music. This suggestion is supported by previous findings in the Stroop test where a dissonant rendition of a Mozart piece, but not the original version, caused a similar increase in conflict cost (Masataka and Perlovsky, 2013). In the case of music, its unique or pleasurable acoustic properties, where an ordered arrangement of different harmonic tones exist (Roederer, 2008), may have prevented such an impairment, by which individuals failed to be concerned or distressed by its presence. Alternatively, the range of valences and tempos present throughout the classical playlist may have resulted in continuing, contrasting alterations to their emotional state (Schmidt and Trainor, 2001; Carpentier and Potter, 2007; Arjmand et al., 2017; Mansouri et al., 2017a), which subsequently summed to no overall effect. In either circumstance, our findings do not support the idea that the perception and processing of classical music can modulate conflict processing.

Despite failing to provide clear recommendations for therapeutic use, our results may help to reconcile earlier comparisons made between music and other non-musical sounds in the context of cognitive task performance. The dichotomy in the effects of listening to the classical playlist and white noise suggests music, in its most general form, can be considered less detrimental to cognitive performance compared to other non-musical sounds. Previous reports, where music has been claimed to equally impair performance compared to noise (Furnham and Strbac, 2002), may instead be explained by emotional modulation related to the tempo and valence of chosen songs, which in the aforementioned study was ‘garage’ music of high intensity/tempo, and the subsequent influence of these modulations on processes involved with performing the cognitive task. This is supported by findings where high tempo music and noise produce near parallel (negative or neutral) effects in task performance, but low tempo music conversely facilitates performance (Cassidy and MacDonald, 2007; Proverbio et al., 2015). However, the directionality of effects induced by these aspects of music on cognitive performance has yet to be consistently established, with studies reporting opposing effects in response to high tempo (Brodsky, 2001; Day et al., 2009; Brodsky and Slor, 2013) and low tempo (Cassidy and MacDonald, 2007; Proverbio et al., 2015; Cloutier et al., 2020) conditions, whilst definitions of valence have varied considerably between studies (e.g., consonant vs. dissonant intervals, major vs. minor chords/scales, positive vs. negative lyrical content).

Moreover, it remains unclear if inter-individual differences may modulate these effects, where sex (Jing et al., 2012; Feizpour et al., 2018) and personality type (Furnham and Strbac, 2002; Dobbs et al., 2011) have been found to differentiate music’s influence in several cognitive tasks. Thus, there is still significant effort to be expended in determining how music can be applied to improve executive control. Despite our results showing classical music has no effect on conflict processing, future studies may consider extending our findings to other, more nuanced musical genres, particularly modern forms of music which can depart significantly from classical composition. It is also crucial to better understand the task-dependent and inter-individual differences between studies assessing changes in tempo and valence, particularly by further uncovering their neural substrate and parameters surrounding their relevant emotional changes.

### White noise increased the conflict cost

In using the Stroop test, we hoped to gain insight into whether white noise can affect performance in a visual-based task assessing a core component of executive control (conflict processing). We found that white noise increased the conflict cost, therefore indicating its continuing presence diminished the ability of participants to perform the relevant functions involved in resolving conflict, which involves a network of prefrontal regions (Botvinick et al., 2001; Kerns et al., 2004; Mansouri et al., 2017b,^2022^; Mansouri and Buckley, 2018). At first, it may be considered that any task-irrelevant information, such as environmental sound, might act as an extra-task distracting feature, engaging parts of the limited cognitive resources that could otherwise be directed toward the current task (Beaman and Jones, 1998; Jones et al., 1999; Elliott and Cowan, 2005; Kerzel and Schönhammer, 2013). However, the failure of music, which was also task-irrelevant and played at the same intensity, to cause similar impairments instead suggests other mechanisms may be responsible.

The prefrontal cortex is highly sensitive to stress (Arnsten, 2009), where despite short-lived levels potentially ameliorating its functioning (Hartley and Adams, 1974; Fehring et al., 2019b), prolonged exposure to negative emotional stimuli, alongside public speaking tasks, has been found to significantly decrease prefrontal activation and impair performance in several of its functional domains (Dolcos and McCarthy, 2006; Alexander et al., 2007; Luethi et al., 2009; Qin et al., 2009). Continuous exposure to loud levels of white noise (≥ 85 dB) has also been used as an acute stressor for behavioural studies in both humans and monkeys, where it has been found to impair higher cognitive function in a manner consistent with observations from other stress-inducing stimuli (Arnsten and Goldman-Rakic, 1998; Hillier et al., 2006; Banis and Lorist, 2012). Whilst such findings may primarily stem from the intensity of the stimulus, it has also been reported that individuals consistently rank various noises (played at a moderate level) lower in terms of emotional preference compared to musical excerpts (Gomez and Danuser, 2004), hence it is likely that continued exposure to moderate levels of white noise might also lead to higher levels of stress and a negative emotional state, albeit to a lesser degree than that observed at higher intensity levels. Subsequently, when considering cognitive task performance and moderate levels of white noise, it is possible there may exist a ‘trade off’ between facilitation caused by stochastic resonance, and impairment arising from a negative response to its presence, where the balance depends not only on the involved brain regions, but also on different factors such as the mode of stimulus presentation and task difficulty.

In this context, distinctions between working memory tasks, where performance involving auditory, but not visual stimuli is improved by white noise (Manan et al., 2012; Bottiroli et al., 2014; Herweg and Bunzeck, 2015; Othman et al., 2019), may arise from the crossmodal nature of stochastic resonance, through which it is posited white noise’s ability to improve tactile, visual, and proprioceptive processing originates from facilitation in multisensory regions such as superior colliculus and posterior parietal cortex (Manjarrez et al., 2007; Lugo et al., 2008). Whereas the linear facilitation in auditory processing regions and upstream activity may be sufficient to overcome, or be greater in magnitude than any impairment resulting from a negative response to the white noise, its more indirect influence on visual processing and subsequent functions may not be of the same degree, and hence fail to significantly improve performance. Findings in healthy children, where performance remained unchanged in an auditory working memory task during white noise exposure (Söderlund et al., 2016), may initially appear to contradict this reasoning, however, such failure may instead likely result from their continuing auditory development, where the ability to accurately separate acoustic stimuli, alongside narrowing of frequency sensitivities, do not reach completion until teenage years (Maxon and Hochberg, 1982; Trehub et al., 1989; Werner, 2007).

Regarding task difficulty, animal and human studies have consistently found that during periods of stress, behavioural responses switch from involving adaptive, albeit slow processes originating from higher order regions such as the prefrontal cortex, to reflexive and rapid processing utilising primarily subcortical regions (Murphy et al., 1996; Lupien et al., 1997; Elliott and Packard, 2008; Luethi et al., 2009). Therefore, the degree by which a given cognitive task requires flexible behaviour and novel decision making, as opposed to repetitive, previously learnt, or solely reflexive responding may also contribute to whether performance is inhibited, or facilitated by exposure to white noise. Previous findings, where performance in tasks that assess an individual’s ability to form stimulus associations or recall previously displayed stimuli is improved by the presence of white noise (Rausch et al., 2014; Herweg and Bunzeck, 2015; Angwin et al., 2017), but not in tasks that require more transient goal-related memory (except for auditory information, as explained above), directed shifting of attention, or complex phonemic construction (Bottiroli et al., 2014; Herweg and Bunzeck, 2015) supports this notion. Our present results, where white noise increased response time in incongruent (high conflict/task load), but not congruent (low conflict/task load) trials may also be interpreted in this context. It is important to note that such distinctions may not apply to ADHD-diagnosed individuals, where improvements have been found in prefrontal domains such as working memory and response inhibition (Helps et al., 2014; Söderlund et al., 2016), likely due to physiological/neurochemical differences as outlined in the authors’ moderate brain arousal hypothesis (Söderlund et al., 2007, 2010, 2016).

Altogether, it is evident that the cognitive influence of white noise is dependent on the abilities/tasks, individuals, and stimulus intensities involved. From our present findings and previous literature, however, only a rudimentary understanding of the parameters by which these factors alter its influence can be proposed. Accordingly, its use in the context of psychiatric care should remain narrowly targetted to particular patient groups where benefit has been clearly described, such as children diagnosed with ADHD (Söderlund et al., 2007, 2010, 2016). Crucial areas for future research include examining how changes in stimulus intensities (outside of signal-to-noise ratios in auditory-based tasks) and cognitive tasks can modulate its influence on behavioural measures, alongside identifying the relevant alterations in underlying physiological processes through functional imaging methods (EEG, fMRI).

### Home-based experiments to examine the effects of contextual factors on cognitive functions

By comparing rule-based biases in the WCST, we found that the results obtained by this home-based study were closely compatible with those obtained within previous laboratory-based studies (Supplementary Figures 2A,B). Recently, the COVID-19 pandemic and related social-distancing measures have forced many researchers to rethink how they conduct research, particularly for behavioural studies which require considerable face-to-face interactions. In view of our results, and complementing previous studies (Semmelmann and Weigelt, 2017), remote testing may provide a viable alternative for researchers faced with these challenges. This can include online testing platforms, as well as the development of offline software that can easily be used by individuals on non-specialised computers. The benefits of remote testing, however, can extend beyond pandemic-related situations. For individuals where laboratory access may be unfeasible, such as those residing in aged care facilities and schools, are mobility-limited, or are geographically isolated, advances in remote testing may foster their inclusion in cognitive and behavioural studies. In the context of Australian healthcare, Aboriginal and Torres Strait Islander individuals are 25% more likely to suffer from psychiatric illness (Australian Bureau of Statistics, 2016a,b), and experience burden from mental and substance use issues at a rate of 2.4 times the overall population (measured through disability-adjusted life years) (Australian Institute of Health and Welfare, 2016). Concurrently, the majority (65%) of aboriginal and Torres Strait islander individuals live away from capital city metropolitans (compared to 25% of Australia’s overall population) (Australian Bureau of Statistics, 2016c), and thus there may exist a significant disparity in their research participation. Similar health inequalities may also be present in other isolated (geographically, health-related, or politically) populations globally, therefore alternative reliable and validated approaches to laboratory-based testing should be considered a viable option for addressing such inequalities, alongside preparing for any potential future disruptions in travel and social contact.

### Within-session learning was not affected by either acoustic condition

Within each daily session, participants’ performance markedly improved from the first to the second stage in both tests. These practice-related, within-session learning effects have been previously reported in the context of other cognitive tasks (Mansouri et al., 2017a; Fehring et al., 2019a,^2022^), and the WCST (Fehring et al., 2019b). We did not find any interactions with task conditions, or acoustic environment in either test, suggesting that task-related learning remained unaffected by classical music and white noise in their respective sessions. Investigations into whether listening to music during study can ameliorate learning processes have generally indicated that when individuals feel positive toward its presence, benefit in a variety of tasks can be derived (Miskovic et al., 2008; Dosseville et al., 2012; Kang and Williamson, 2013). Our findings, where classical music of mixed valence (and therefore unlikely to consistently appease listeners) did not affect within-session learning, further supports the idea that these reported improvements arose from participants’ subjective enjoyment of the music, and not from other aspects of its perception and processing. Furthermore, the similar failure of white noise suggests that the apparent task-dependencies of its cognitive effects extend to related learning processes, where our results contrast findings that white noise augments performance improvements over consecutive testing stages (within the same day) in a semantic memory task (Angwin et al., 2017).

### Limitations

In this study, all participants were young undergraduate students within a limited age range (18–29 years old), and hence were a reasonably homogenous cohort. Accordingly, our findings cannot be directly applied to individuals outside this age range, where further research may be required. Since participants completed the tests from their homes, there may have been significant differences in the background noise levels (e.g., traffic levels). Although they were instructed to perform the tests at the quietest possible time of day, and our design was repeated-measures, variations in environmental noise may have impacted participants’ test performance and perception of acoustic stimuli. Further studies may serve to validate our results in more controlled laboratory environments. Participants also used different mediums for playing the acoustic stimuli (e.g., headphones, earphones, speakers), and hence the quality of acoustic stimuli may have fluctuated between individuals. Whilst such alterations to quality would have been constant for each participant because of the repeated-measures design, it is possible they may have led to different effects between individuals. Future home-based studies may endeavour to provide participants the same acoustic medium to account for such possibilities. Lastly, there was no direct measure of biological activity to accompany the behavioural data in this study. Therefore, we could not provide direct evidence for the potential physiological underpinnings of our observed results, particularly for whether the dissociable effects of music and white noise observed in this study did in fact arise from differing emotional reactions, or stress responses. Future studies should consider attempting to validate (or disprove) our hypothesis with the inclusion of such measures.

## Implications of our findings

Our findings indicate that compared to silence, background classical music and white noise differentially affect performance in the Stroop test, where classical music has no influence, and white noise increases the response time difference between congruent and incongruent trials (conflict cost). Such effects cannot be explained by white noise acting as task-irrelevant information and thus occupying limited cognitive resources (Beaman and Jones, 1998; Jones et al., 1999; Elliott and Cowan, 2005; Kerzel and Schönhammer, 2013), as music was played at a similar intensity, yet did not produce a similar impairment. Therefore, it is possible participant’s may have experienced a negative emotional response to the presence of the white noise, hence impairing their ability to resolve conflict, whilst it is unclear whether the presence of certain pleasurable acoustic qualities, or fluctuating alterations to their emotional state may have prevented a similar response to the music.

The absence of effect in the music condition suggests varied (no specific tempo, valence) classical music may not be an appropriate acoustic medium for improving conflict processing, but it is important to note these findings cannot be extended to other, more nuanced or modern genres of music, which requires further investigation. Meanwhile, the impairment caused by white noise indicates its cognitive effects may be nuanced and particularly sensitive to differences between tasks and individuals. In particular, these adverse effects highlight the necessity for further research on white noise’s interactions with cognitive functions before it can be recommended for addressing other symptoms of neuropsychological disorders (Kaneko et al., 2013; Lin et al., 2018). Non-human primate models may be suitable for continuing such investigations, given the similarity of our present results and those reported in one of our previous studies (Zarei et al., 2019). Lastly, the correspondence of rule biases within the WCST between home-based, and laboratory-based experiments (Mansouri et al., 2020a) indicates remote testing may be a reliable avenue for psychophysical testing when in-person contact cannot be achieved.

## Data availability statement

The original contributions presented in the study are included in the Supplementary material, further inquiries can be directed to the corresponding author/s.

## Ethics statement

The studies involving human participants were reviewed and approved by Monash Human Ethics Committee. The patients/participants provided their written informed consent to participate in this study.

## Author contributions

AP conducted the study, analysed the data, and wrote the manuscript. ZH and RS participated in data collection, analysis, and writing the manuscript. DF and FM participated in analysis and writing the manuscript. All authors contributed to the article and approved the submitted version.
