# Supplementary material for: Dissociable effects of music and white noise on conflict-induced behavioral adjustments
Source: Front Neurosci. 2022 Aug 17;16:858576. doi: 10.3389/fnins.2022.858576 (PMC9429995; doi:10.3389/fnins.2022.858576)
Supplement: Supplementary file 1 [file Data_Sheet_1.docx]

**Supplementary Material**

**Appendix 1. Wisconsin card sorting test**

### **1.1 Methods: PsyToolkit Wisconsin card sorting test**

In this WCST analogue (Supplementary figure 1), participants are directly presented with one sample card and four test cards. Cards can vary between number of items, shape, and colour, which are the rules used for matching. Participants are instructed to match the sample card to one of the test cards according to the relevant rule by clicking the matching card with their mouse. Correct responses are indicated by a green text box displaying ‘Good!’, whilst incorrect responses result in a white text box displaying ‘WRONG!’, both appearing underneath the participants’ selection. After 1000ms, this feedback is removed and the following trial commences. Failure to respond within 10,000ms (10s) causes a yellow text box with ‘Too Slow’ written in red to appear, also being removed after 1000ms. Each rule was relevant for a block of 10 trials, regardless of accuracy, before being changed unannounced to participant. In this cohort (whom were young and neurologically normal), we observed that following a few errors, participant accuracy rapidly increased and often reached a near maximal level by the end of each block. In each testing stage, participants completed six blocks of ten trials. After completing the first three blocks (one of each rule), a new set of 3 blocks, and thus a different order of rules, was run.

### **1.2 Results: Matching rule influenced performance**

A multifactorial repeated-measures ANOVA [Practice (first/second stage of testing, within-subject factor) x Rule (colour/shape/number, within-subject factor) x Sound (music/noise/silence, within-subject factor) x Sex (female/male, between-subject factor)] applied to mean normalized response time revealed a significant main effect of Practice (F(1,64) = 48.25; *p < 0.001*, η_p_^2^ = 0.442): response time shortened from the first to second stage of testing within the same session (within-session learning). Importantly, the main effect of Rule was highly significant (F(2,128) = 91.93; *p < 0.001*, η_p_^2^ = 0.60): response time was different depending on the relevant rule (Supplementary figure 2A). The main effect of Sound was not significant (F(2,128) = 0.64 ; *p = 0.53*): response time in the WCST was not influenced by the background acoustic condition. Using non-normalized response time values in the same multifactorial repeated-measure ANOVA produced comparable results, with significant main effects found for Practice (F(1,64) = 37.48; *p < 0.001*, η_p_^2^ = 0.38) and Rule (F(2,128) = 69.93; *p < 0.001*, η_p_^2^ = 0.53), and an insignificant main effect for Sound (F(2,128) = 0.22 ; *p = 0.80*).

To further confirm that there was a bias to colour dimension over shape, a planned pairwise comparison (paired two-tailed t-test) was conducted to compare the normalised response time between colour and shape blocks. The difference in response time between colour and shape rules was significant (t(63) = 12.81; *p < 0.001*). This finding replicated what we have detected in laboratory-based studies regarding the difference between colour and shape rules, and confirmed the sensitivity and reliability of the home-based studies (Supplementary figure 2A-B). An identical paired two-tailed t-test using raw mean response time replicated the results (t(63) = 10.97; *p < 0.001*).

**Appendix 2. Correspondence between human and non-human primate studies**

Whilst the use of non-human primates for investigating interactions between executive control and auditory stimuli, particularly music, has been scarce, there exists evidence to suggest that some monkey species may share human’s positive affiliation for certain acoustic characteristics such as rhythm (Mingle et al. 2014) and harmony (Sugimoto et al. 2010), and hence their inclusion in the field may be justified. Similar to our present findings (but for a different cognitive function), differential effects between music and white noise on working memory performance have been reported in two separate macaque monkey studies, but surprisingly in opposite directions (Carlson et al. 1997, Zarei et al. 2019). In the first of these studies (Carlson et al. 1997), monkeys performed a delayed response task, where they had to recall the location of a reward placed in one of two wells after a blinded delay. Concurrent exposure to a Mozart piece was found to impair the accuracy when task difficulty was increased (longer delay), whilst white noise instead facilitated their performance. Our more recent study (Zarei et al. 2019), which involved a modified delayed matching to sample task, required monkeys to hold information of a visual sample during a delay period, and then select and touch the test item that matched the sample. Visual stimuli, which were used as the sample and test items, varied in their emotional content and included positive (e. g. macaque children, parental caring), negative (e. g. predators), or neutral (e. g. house, car) images. We found that macaques’ performance was adversely affected by the emotional content of the stimuli, and appeared as a longer response time for negative and positive stimuli in both the silence and classical music conditions. White noise, however, significantly interacted with the emotional content of the stimuli and abolished the effects of emotional stimuli, increasing response time in the neutral condition to similar levels.

Remarkable, is the homology of our present results and those in our macaque study (Zarei et al. 2019), where in both studies classical music left performance unaffected, but white noise caused impairments in a condition-dependent manner. Together, these findings indicate that across species, white noise affects cognitive performance in a discreet and interactive manner, where performance in some domains, but not others (even within the same task) is affected, and further clarifies why contrasting effects have been reported in various contexts, tasks, and species. Moreover, this correspondence adds support for the use of non-human primate models in uncovering the neural basis of the influence of background acoustic conditions on cognitive functions, where more detailed neurobiological techniques may be employed. Implications from findings in non-human primates may also extend past exploring therapeutic use for humans, with the use of auditory stimuli such as music and white noise steadily increasing within animal laboratories (Patterson-Kane & Farnworth 2006).

**Appendix 3. Classical music playlist**

List of songs included in music condition. This playlist can be accessed at: (https://archive.org/details/100ClassicalMusicMasterpieces)

1. Purcell – Trumpet Tune and Air (1685)
2. Pachelbel – Canon in D (1698)
3. Bach – Toccata in D Minor (1709)
4. Handel – Water Music, Suite No. 2 in D (1717)
5. Bach – Brandenburg Concerto No. 3, 1^st^ Movement (1721)
6. Bach – Minuet and Badinerie, From Orchestral Suite No. 2 in B Minor (1721)
7. Vivaldi – The Four Seasons, Spring (1725)
8. Bach – Air, From Orchestral Suite No.3 in D (1727)
9. Vivaldi – Mandoline Concerto in C, RV 425 (1729)
10. Albinoni & Adagio – Classical (1730)
11. Bach – Oboe Concerto in D Minor, 2^nd^ Movement (1731)
12. Vivaldi – Flute Concerto in G Minor ‘La Notte’, VI. Allegro (1731)
13. Bach – Sinfonia in G, From Christmas Oratorio (1734)
14. Handel – Largo, From Xerxes (1734)
15. Handel – Concerto Grosso in A Minor Op.6 No. 4 (1739)
16. Handel – Hallelujah, From Messiah (1742)
17. Handel – Arrival of the Queen of Sheba, From Solomon (1749)
18. Gluck – Dance of the Blessed Spirits, From Orpheus and Eurydice (1762)
19. Mozart – Violin Concerto No. 3 in G, 1^st^ Movement (1775)
20. Mozart – Violin Concerto No. 5 in A, 2^nd^ Movement (1775)
21. Mozart – Serenata Notturna (1776)
22. Mozart – Flute Concerto No. 2 in D, 2^nd^ Movement (1778)
23. Mozart – Rondo Alla Turca, From Piano Sonata in A (1778)
24. Mozart – Horn Concerto No. 3 in E Flat, 2^nd^ Movement (1783)
25. Mozart – Piano Concerto No. 21 in C, 2^nd^ Movement “Elvira Madigan” (1785)
26. Mozart – Piano Concerto No. 23 in A, 1^st^ Movement (1786)
27. Mozart – The Marriage of Figaro, March (1786)
28. Mozart – The Marriage of Figaro, Overture (1786)
29. Mozart – Don Giovanni, Overture (1787)
30. Mozart – Eine Kleine Nachtmusik, 1^st^ Movement (1787)
31. Mozart – Symphony No. 40, 1^st^ Movement (1788)
32. Hadyn – Symphony No. 94, “Surprise” 2^nd^ Movement (1791)
33. Mozart – Clarinet Concerto in A, 2^nd^ Movement (1791)
34. Mozart – The Magic Flute, Overture (1791)
35. Beethoven – Minuet in G (1796)
36. Haydn – Trumpet Concerto in E Flat, 3^rd^ Movement (1796)
37. Haydn – Emperor’s Hymn, From String Quartet in C (1797)
38. Beethoven – “Moonligh” Sonata, 1^st^ Movement (1801)
39. Beethoven – Symphony No. 5, 1^st^ Movement (1808)
40. Beethoven – Fur Elise (1810)
41. Schubert – German Dance No. 1 (1811)
42. Weber – Der Freischutz, Overture (1820)
43. Rossini – The Barber of Seville, Overture (1821)
44. Schubert – Symphony No. 8 in B Minor, “Unfinished” (1822)
45. Schubert – Ballet Music in G, From Rosamunde (1823)
46. Schubert – Ave Maria (1825)
47. Mendelssohn – Symphony No. 4 in A, “Italian”, 1^st^ Movement (1833)
48. Chopin – Polonaise in A, Op. 40 No. 3, “Military” (1838)
49. Schumann – Traumerei (1838)
50. Mendelssohn – Spring Song (1841)
51. Verdi – Nabucco, Overture (1842)
52. Mendelssohn – Wedding March, From A Midsummer Night’s Dream (1843)
53. Mendelssohn – Violin Concerto in E minor, 2^nd^ Movement
54. Wager – Tannhäuser, Arrival of the Guests at Wartburg (1845)
55. Suppe – Poet and Peasant (1846)
56. Lizt – Hungarian Rhapsody No. 2 (1847)
57. Lizt – Liebestraum No. 3 in A Flat (1847)
58. Schumann – The Merry Peasant (1848)
59. Wagner – Lohengin, Prelude to Act 3 (1850)
60. Verdi – La Traviata, Prelude to Act 1 (1853)
61. Lizt – Les Preludes (1854)
62. J. Straus II – Tritsch Tratsch Polka (1858)
63. Offenbauch – Orpheus in The Underworld (1858)
64. Rubinstein – Melody in F (1858)
65. Verdi – The Force of Destiny, Overture (1862)
66. Grieg – I Love You (1864)
67. Offenbauch – Barcarolle, From The Tales of Hoffmann (1864)
68. Brahms – Waltz (1865)
69. Smetna – The Bartered Bride, Overture (1866)
70. Suppe – Light Cavalry, Overture (1866)
71. J. Strauss II – The Blue Danube (1867)
72. Brahms – Cradle Song (1868)
73. Delibes – Notturno, From Coppelia (1870)
74. Wagner – Ride of The Valkyries, From The Valkyrie (1870)
75. Bizet – L’Arlesienne (1872)
76. Bizet – Les Toreadors, From Carmen (1875)
77. Smetana – The Moldau (1875)
78. Tchaikovsky – Piano Concerto No. 1 in B Minor Flat Minor, 1^st^ Movement (1875)
79. Grieg – Morning, From Peer Gynt (1876)
80. Tchaikovsky – Marche Slave, Op. 31 (1876)
81. Wagner – Siegfried’s Death and Funeral March, From Twilight of The Gods (1877)
82. Tchaikovsky – Polonaise, From Eugene Onegin (1878)
83. Dvorak – Songs My Mother Taught Me (1880)
84. Dvorak – Slavonic Dance No. 2 (1886)
85. Grieg – The Last Spring (1886)
86. Rimsky-Korsakov – Alborado, From Capriccio Espagnol (1887)
87. J. Straus II – Emperor Waltz (1889)
88. Tchaikovsky – The Sleeping Beauty, Introduction (1889)
89. Tchaikovsky – Waltz of The Flowers, From The Nutcracker (1892)
90. Dvorak – Symphony No. 9, From The New World (1893)
91. Dvorak – Humoresque (1894)
92. Massenet – Meditation, From Thais (1894)
93. R. Strauss – Also Sprach Zarathustra, Fanfare (1896)
94. J. Strauss II – Vienna Blood, Waltz (1899)
95. Sibelius – Finlandia (1899)
96. Rimsky-Korsakov – Dance of The Bumble Bee (1900)
97. Elgar – Pomp and Circumstance, March No. 1 (1901)
98. Mahler – Symphony No. 5, Adagietto (1902)
99. Sibelius – Valse Triste (1903)

100. Ravel – Bolero (1928)

**Supplementary Figure**

**
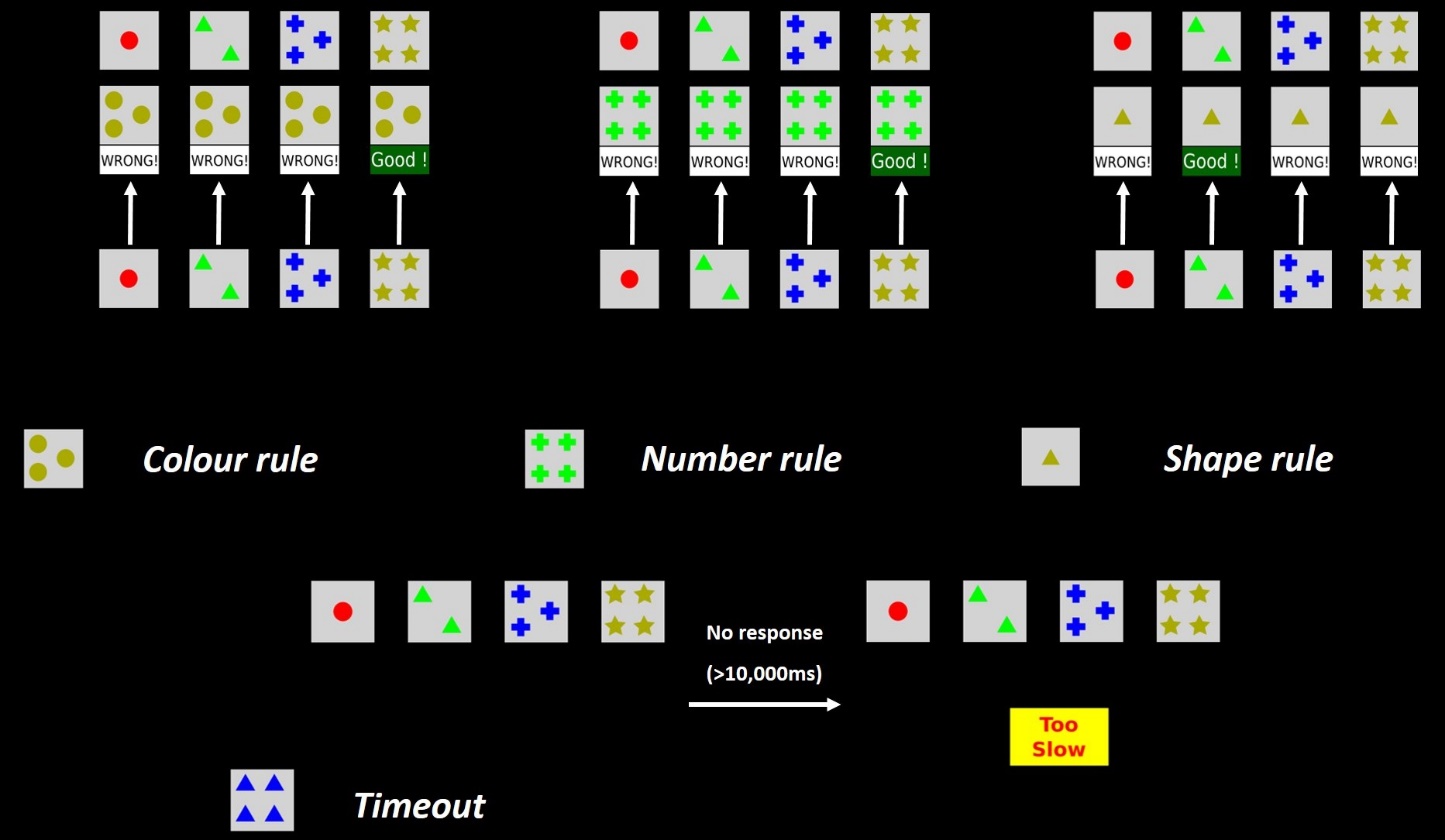
**

***Supplementary figure 1. Schematic representation of the experimental paradigm in the PsyToolkit Wisconsin card sorting test.*** *Trials begin with the presentation of one sample card and four test cards. A correct response is indicated by a green text box displaying ‘Good!’ appearing underneath the selected card, whilst an incorrect response is indicated by a white text box displaying ‘WRONG!’ underneath the selection. If no response is given after 10,000ms, a yellow text box with ‘Too Slow’ in red appears in the middle of the screen.* (Stoet 2010, 2016)


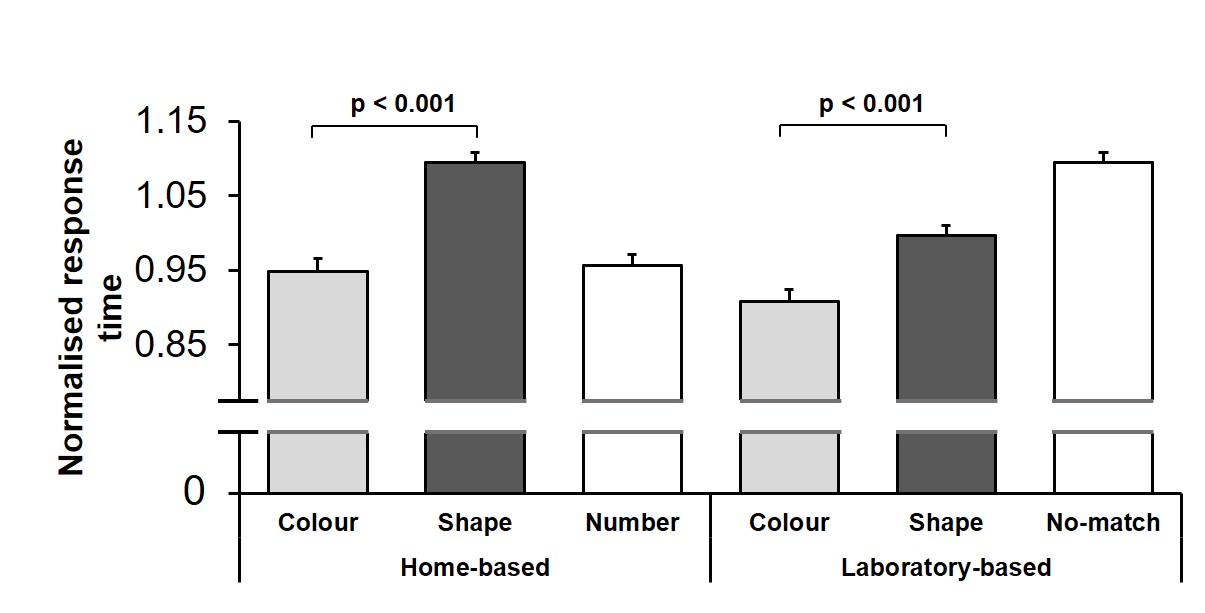


***Supplementary figure 2. Bias to colour-matching in the Wisconsin card sorting test was replicated in home-based experiment.*** ***(A)*** *Mean normalized response time is shown for each matching rule in home-based (current study), and* ***(B)*** *laboratory-based (Mansouri et al. 2020b) experiments. In both experiments, response time was significantly shorter in colour-matching trials compared to shape-matching trials. The third matching rule was different between tests (number for home-based, and no-match for laboratory-based), but did not affect the observed dimensional bias. Error bars represent standard error of the mean (SEM).*
