# Supplementary figures and images for: Dissociable effects of music and white noise on conflict-induced behavioral adjustments
Source: Front Neurosci. 2022 Aug 17;16:858576. doi: 10.3389/fnins.2022.858576 (PMC9429995; doi:10.3389/fnins.2022.858576)

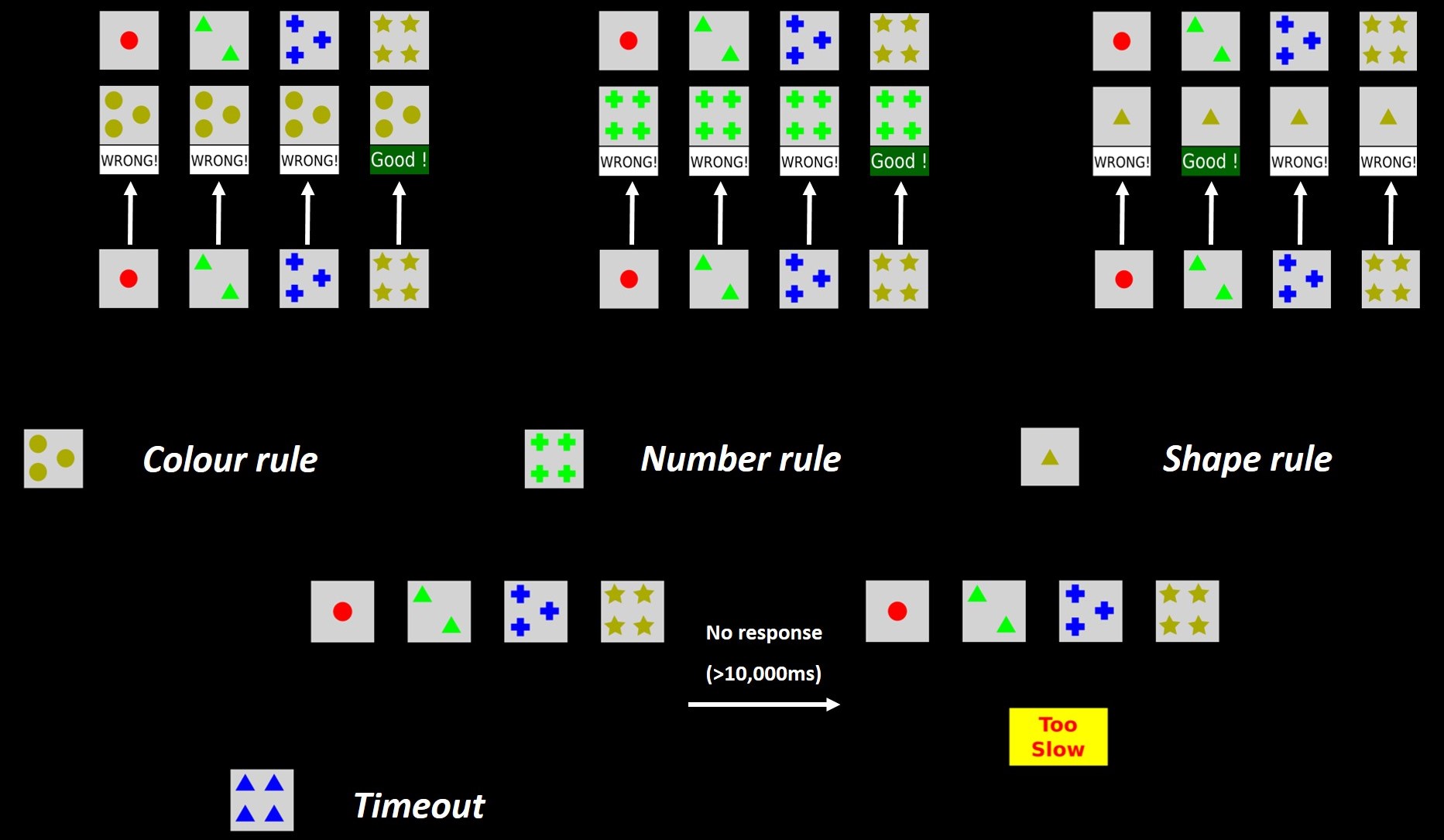

Supplement: Supplementary file 2 [file Image_1.JPEG]

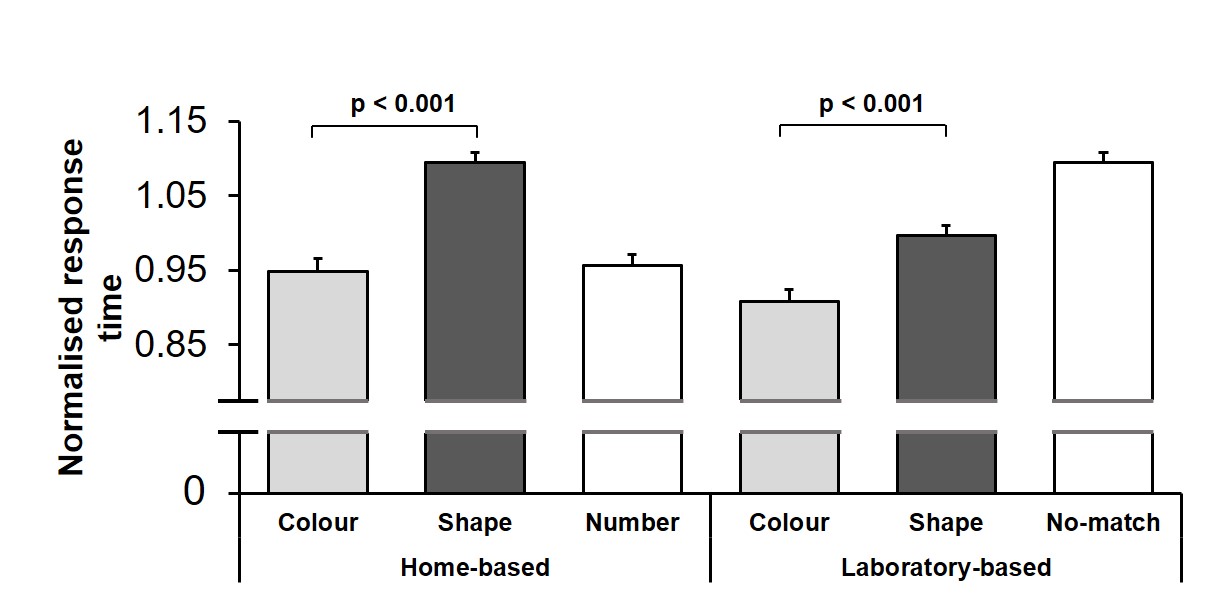

Supplement: Supplementary file 3 [file Image_2.jpg]
